# MRI and Targeted Biopsy Essential Tools for an Accurate Diagnosis and Treatment Decision Making in Prostate Cancer

**DOI:** 10.3390/diagnostics11091551

**Published:** 2021-08-27

**Authors:** Suraj Samtani, Mauricio Burotto, Juan Carlos Roman, Daniela Cortes-Herrera, Annerleim Walton-Diaz

**Affiliations:** 1 Clinical Research Center, Bradford Hill, Santiago 8420383, Chile; suraj_rsb@hotmail.com (S.S.); Mauricioburotto@gmail.com (M.B.); 2 Fundacion Chilena de Inmuno Oncologia, Santiago 8420383, Chile; 3 Oncología Médica, Clinica Universidad de los Andes, Santiago 7620157, Chile; 4 Urofusion Chile, Santiago 7500010, Chile; dr.jcrs@gmail.com (J.C.R.); daniela.cortes@urofusion.cl (D.C.-H.); 5 Servicio de Urologia, Instituto Nacional del Cancer, Santiago 8380455, Chile; 6 Departamento de Oncologia Básico-Clinico Universidad de Chile, Santiago 8380455, Chile

**Keywords:** prostate cancer, MRI, artificial intelligence, diagnosis, active surveillance

## Abstract

Prostate cancer (PCa) is one of the most frequent causes of cancer death worldwide. Historically, diagnosis was based on physical examination, transrectal (TRUS) images, and TRUS biopsy resulting in overdiagnosis and overtreatment. Recently magnetic resonance imaging (MRI) has been identified as an evolving tool in terms of diagnosis, staging, treatment decision, and follow-up. In this review we provide the key studies and concepts of MRI as a promising tool in the diagnosis and management of prostate cancer in the general population and in challenging scenarios, such as anteriorly located lesions, enlarged prostates determining extracapsular extension and seminal vesicle invasion, and prior negative biopsy and the future role of MRI in association with artificial intelligence (AI).

## 1. Introduction

Prostate cancer is the most frequent cancer worldwide in men. Although it is usually considered a slow growing tumor, it remains the third cause of cancer death in the male population. According to Globocan statistics approximately 1,414,259 new cases are diagnosed every year worldwide. The lifetime probability of a man developing PCa is 1 in 9 and the number of estimated deaths caused by PCa in the world during 2020 was 375,304. The regions with higher incidence of PCa are Northern Europe, North America, Caribbean, Australia, and New Zealand [1].

Historically, when PCa was suspected, diagnosis was based on an elevated prostate-specific antigen (PSA), abnormal digital rectal examination, and transrectal ultrasound (TRUS) images, all known to have low sensitivity and specificity for cancer diagnosis. Then a systematic TRUS-guided biopsy was indicated that sampled the main six areas of the peripheral zone of the prostate. However, due to its random nature, this approach has several limitations, such as overall low cancer detection rates as well as a high percentage of detection of indolent prostate cancer. This can lead to overtreatment of a group of patients. During the past few years, the use of MRI as a diagnostic tool has gained momentum with increasing roles in diagnosis, staging, treatment decision, and follow-up of prostate cancer. This review summarizes the importance of MRI in prostate cancer history, its actual recommendations in clinical practice, and the future role of MRI in combination with artificial intelligence.

### History of Prostate Biopsy from Sextant Biopsy to MRI Guided Biopsy

In 1971, Damadian et al. first explored the application of MRI in the diagnosis of cancer in six normal tissue samples and two malignant solid tumors in murine animals. Malignant tissues could be differentiated according to the T1 and T2 relaxation times, as such parameters were outside the range of values when compared with the normal tissues [2]. Furthermore, in 1982, Steyn and Smith reported their initial findings for prostate MRI performed on 25 men in Aberdeen with use of a four-coil, air-cored magnetic ring with a static magnetic field. It first described the anatomical extent and pathological nature of prostatic lesions [3]. It was in 1991 that Narayan et al. assessed the ability of 31phosphorus transrectal magnetic resonance spectroscopy to characterize normal human prostates as well as abnormal prostates, including malignant neoplasms. Different metabolite ratios were detected between groups, which suggested that transrectal 31P magnetic resonance spectroscopy could characterize metabolic differences between the normal and malignant prostate tissue [4]. Initially, MRI for prostate cancer detection and staging used T2 weighted images, diffusion-weighted imaging (DWI), contrast enhancement, and spectroscopy. However, the cost and logistics for using all these parameters has made simple algorithms with use of abbreviated protocols a more popular approach. As of today, most centers worldwide still perform T2 weighted images, DWI, and contrast enhancement. The most recent version of prostate imaging reporting and data system (PI-RADS v.2.1) provides recommendations for interpretation of results observed in MRI in the diagnosis of prostate cancer to standardize inter-reader variability [5].

## 2. MRI Guided Biopsy Techniques

In terms of MRI guided biopsy for suspicious lesions, various sampling techniques are used to secure targeted biopsies, i.e., in-bore MRI-guided biopsy, cognitive biopsy, and MRI-TRUS fusion-guided biopsy. Each technique has strengths and limitations. Cognitive MRI targeted biopsy is a less expensive and time-consuming biopsy and does not require software-based platforms but needs operator expertise on MRI lesion localization. In-bore MRI-guided biopsy provides direct visualization of lesions, with fewer cores needed. However, the length of the procedure is longer, which increases the cost of the technique. Table 1 shows a comparison of prostate biopsy techniques.

A meta-analysis compared three different techniques for MRI targeted biopsy (in-bore MRI target biopsy (MRI-TB) [2], MRI-transrectal ultrasound fusion (FUS-TB), and cognitive registration (COG-TB). MRI-TB had higher detection rates of clinically significant PCa compared with TRUS-GB (RR 1.16 (1.02–1.32)), and a lower yield of insignificant PCa (RR 0.47 (0.35–0.63)). For overall PCa detection there was no significant advantage of MRI-TB compared with FUS-TB (*p* = 0.13), and neither for FUS-TB compared with COG-TB (*p* = 0.11) [6]. Guidelines as of 2021 encourage the use of MRI-GB to increase the accuracy of significant prostate cancer detection, suggesting that the technique employed should consider costs and the centers expertise [7,8].

## 3. Impact of MRI in Diagnosis, Staging, Treatment Decision Making, and Follow-Up

### 3.1. Diagnosis of Prostate Cancer

The use of MRI in detection of PCa has been validated in different clinical settings. PROMIS and PRECISION trials reported the diagnostic accuracy of the use of MRI in detection of PCa. In the PROMIS study, 740 men were enrolled and 576 men were randomized to multiparatric MRI (mpMRI) targeted biopsy group versus standard biopsy. Clinically significant cancer was detected in 40% in the MRI-TB group, compared to 26% in the standard-biopsy group. Furthermore, mpMRI with or without targeted biopsy was non inferior to standard biopsy, and the 95% confidence interval indicated the superiority of this strategy over standard biopsy [9].

The PRECISION trial showed that mpMRI was more accurate than TRUS-biopsy for PCa detection. This trial randomized 500 patients with elevated PSA and suspicion of PCa to either mpMRI or standards biopsy. For those men who underwent mpMRI if they were diagnosed with a suspicious lesion ≥ 3, a targeted biopsy of the lesions was performed. Prostate cancer was detected in 95 men (38%) in the MRI-targeted biopsy group, as compared to 64 of 248 (26%) in the standard-biopsy group (*p* = 0.005). In terms of both sensitivity (93% vs. 48%) and negative predictive value (89% vs. 74%) mpMRI was superior to the standard approach and could identify a quarter of men who might safely avoid an unnecessary biopsy. Additionally, the PRECISION study reported that mpMRI had a 29% detection rate of clinically significant (CS) PCa (defined as Gleason Score ≥ 3 + 4 = 7) for PIRADS 3 lesions, 40% for PIRADS 4, and 31% for PIRADS 5 [10]. Table 2 summarizes the findings of both PROMIS and PRECISION trial.

Similarly, Ahdoot et al. studied the cancer detection rates in 2103 men undergoing mpMRI-targeted biopsy and standard biopsy. High-grade clinically significant PCa detection rates were higher in the group of mpMRI-targeted biopsy compared with standard biopsy [11]. 

### 3.2. The Role of MRI in Population of Patients with a Rising PSA and Prior Negative Systematic TRUS-Guided Biopsy

mpMRI has proven to be a helpful tool in assessing patients with rising PSA and prior negative standard biopsy in which PCa is needed to be ruled out as a diagnosis. Abd-Alazeez et al. reported the performance of mpMRI specifically in this subgroup of patients. Overall cancer detection rate was 63% and mpMRI accuracy for detection of clinically CS PCa showed a sensitivity of 76%, and negative predictive value of 79% [12]. In 71 patients with rising PSA and history of two or greater negative biopsies, mpMRI-targeted biopsy detected prostate cancer in 59% of patients and in this subgroup 93% of cases were considered CS PCa [13].

The use of MRI in patients with prior negative biopsy has also been studied with interesting results by Vourganti et al. who assessed cancer detection rates for this group of patients when using mpMRI and FUS-TB in 195 men with previous negative biopsies. The authors reported that 73 (37%) men were found to have cancer and high-grade cancer (Gleason score ≥ 8) was detected in 21 (11%) of them. Furthermore, 55% of high-grade cancers were missed by standard transrectal ultrasound biopsy. Pathological upgrading occurred in 28 men (38.9%) as a result of mpMRI- FUS-TB vs. standard transrectal ultrasound biopsy [14]. Another study performed in Netherlands randomized 665 patients with prior negative biopsy to mpMRI using Prostate Imaging Reporting and Data System (PIRADS) version 2 and mpMRI targeted biopsy if imaging demonstrated PIRADS ≥3 lesions. The overall detection rate using targeted biopsy was higher than the reported in the literature for standard systematic TRUS-guided biopsy. Additionally, there were no significant differences in the detection rates of overall prostate cancer when comparing different techniques for targeted biopsy (FUS-TB 49%, COG-TB 44%, and MRI-TB 55%, *p* = 0.4) [15] Results are summarized in Table 3.

MRI has also shown to improve cancer detection in patients with no prior biopsy. A meta-analysis including 29 studies with 13,845 patients compared the use of mpMRI and targeted biopsy to standard biopsy. 15% higher rate of any PCa diagnosis was reported in the mpMRI targeted biopsy group. Diagnoses of clinically significant and high-grade PCa were more common in the mpMRI imaging targeted biopsy group while there was no difference in diagnosis of clinically insignificant PCa [16].

Another important contribution of MRI is to be able to differentiate between benign and malignant tissue. In terms of factors predicting for benign pathology on mpMRI, Troung et al. developed a nomogram that considered the use of parameters such as age, prostate-specific antigen, prostate volume, and PIRADS score to predict benign pathology on mpMRI. The authors described the results with 2 different thresholds: cutoff 0.7 and 0.4. With a cutoff probability of 0.4, 36.5% of patients would have avoided unnecessary biopsy. However, 6.3% of CS PCa would have been missed. When the cutoff of ≥0.7 was used, 21.4% of patients would have avoided an unnecessary biopsy, and CS PCa would have been missed in only 1.4% of cases [17].

### 3.3. MRI in Unsual Clinical Settings

#### 3.3.1. Anteriorly Located Lesions

Increasing evidence supports the use of mpMRI for anteriorly located lesions which are usually underdiagnosed. These lesions are often missed by conventional standard TRUS-guided biopsy given their anterior location is not easily reached by conventional techniques. In a study by Volkin et al. of 499 patients undergoing MRI-TRUS fusion-guided biopsy, 162 patients had anterior lesions. PCa was detected in 121 anterior lesions (50.2%) identified on mpMRI and 40.2% of these lesions were diagnosed by targeted biopsy compared with 25% diagnosed by the standard TRUS-guided biopsy approach [18].

Another trial included 39 patients with previous negative TRUS-guided biopsy who underwent prostate mpMRI including DWI. Patients with a suspicious anterior lesion on mpMRI underwent targeted anterior gland TRUS-guided biopsy with cognitive fusion technique. Anterior gland prostate adenocarcinoma was diagnosed in 46.2% on targeted cores, and CS PCa was diagnosed in 13 patients (33.3%). Biopsies were positive in 90% of patients with overall PIRADS 5 lesions, and 33% of patients with PIRADS scores of 4 [19]. These studies support the use of mpMRI in cases in which patients present with an elevated prostate-specific antigen and there is suspicion of presence of anterior lesions.

#### 3.3.2. Enlarged Prostates

Standard systematic TRUS-guided biopsy cancer detection rates decrease with increasing prostate volume. In this setting, mpMRI-targeted biopsy appears to increase the accuracy of PCa detection. MRI FUS-TB represents a promising solution for patients with suspicion of PCar and an enlarged prostate. In a trial of 649 patients with a mean whole prostate volume of 58.7 cc the overall cancer detection rate of the MRI FUS-TB was 55%. For prostates < 40 cc the detection rate was 71.1% compared to 57.5% for standard systematic TRUS-guided biopsy, 46.9%, 46.9% 33.3%, 36.4%, and 30.4% for glands 40 to 54.9, 55 to 69.9, 70 to 84.9, 85 to 99.9, 100 to 114.9, and 115 cc or greater, respectively. Inverse association of mpMRI volume with PCa detection, controlling for age and prostate-specific antigen was also reported [20]. Similarly, Gorski et al. found a detection rate of CS PCa of 44% with mpMRI-targeted biopsy in patients with a prostate volume greater than 40 cm^3^. Detection rate was 77% for prostate glands less than 30 cm^3^, and 61%, 47% and 34% for glands 30 to less than 38.5, 38.5 to less than 55, and 55 to 160 cm^3^, respectively (*p* = 0.001) [21]. These studies suggest an important role for MRI targeted biopsy in patients with enlarged prostates who when undergoing systematic TRUS-guided biopsy tend to be underdiagnosed.

### 3.4. Use of MRI for Determining Extracapsular Extension and Seminal Vesicle Invasion

The role of this imaging technique has expanded beyond detection of cancer, including staging of tumors with evaluation of extracapsular extension (ECE) and seminal vesical invasion (SVI), as well as improved characterization of possible candidates for active surveillance. Accurate staging is one of the fundamentals in oncology in order to determine the best therapeutic alternative for patients. mpMRI has demonstrated high specificity and negative predictive value for predicting pathologic ECE and SVI. In a trial of 79 patients that were studied with mpMRI, 28% patients had ECE, 5% SVI, and 4% lymph node involvement (LNI) in mpMRI. Table 4 summarize sensitivity, specificity, accuracy, positive predictive value (PPV), and negative predictive value (NPV) of mpMRI for predicting ECE and SVI [22,23].

Tools such as Memorial Sloan Kettering Cancer Centre and Partin tables have been used to predict ECE or SVI. Recently a nomogram including mpMRI parameters and mpMRI-targeted biopsy was developed. Performance of the nomogram in comparison with the Memorial Sloan Kettering Cancer Center (MSKCC) model and Partin tables, was evaluated. Regarding ECE prediction, the nomogram showed high discrimination (71.8% vs. 69.8%, *p* = 0.3 and 71.8% vs. 61.3%, *p* < 0.001). Performance of the nomogram with regards to SVI was comparable in terms of discrimination (68.5% vs. 70.4% vs. 67.8%, *p* ≥ 0.6), and a net benefit for probability threshold above 7.5% validating a nomogram predicting ECE and SVI in patients diagnosed with mpMRI-targeted biopsy [24].

## 4. Role of MRI in Active Surveillance

Active surveillance is considered an alternative for patients with low-risk prostate cancer. Overtreatment of this group of patients leads to important physical, mental, and economic burden which can be avoided with careful monitoring of this subgroup of patients [25,26,27,28,29]. Klotz et al. in the pre mpMRI era initially reported the results of a prospective trial of 450 patients who underwent active surveillance describing an overall survival of 78.6% for patients opting for this treatment strategy with a median follow-up of 6.8 years [30].

In the mpMRI era, the use of MRI in active surveillance protocols for patients diagnosed with low and intermediate risk PCa has been validated and incorporated to international guidelines. In a single center retrospective study of 133 men with a mean PSA level of 6.73 ng/mL, 40.6% of patients harbored Gleason 3 + 3 and mp MRI demonstrated a sensitivity of 93%, positive predictive value of 57%, and overall accuracy of 92% for identifying candidates for active surveillance based on findings at radicalprostatectomy [31]. Futhermore, in a trial of 50 patients, MRI FUS-TB was superior to standard biopsy for detecting tumor burden greater than 50 mm, reflecting better overall disease burden and improving risk stratification among candidates for active surveillance [26,32].

The ASSIST trial randomized 273 men with low-grade PCa eligible for active surveillance to mpMRI with standard plus targeted biopsy versus standard biopsy alone. After confirmatory biopsy 101 (76%) men in the non-MRI arm and 98 (77%) continued on active surveillance. Median follow-up was two years. This trial demonstrated that mpMRI before targeted biopsy resulted in fewer men with active surveillance failure (19% vs. 35%), and there were fewer men with CS PCa detected at two years after follow-up biopsy in the MRI arm (9.9% vs. 23%) thus confirming the role of mpMRI in men with PCa undergoing this treatment approach. No significant differences in progression free survival (PFS) and radical intervention were documented [33].

MRI guided biopsy has also been studied to determine the risk of pathologic upgrading in patients on active surveillance on confirmatory biopsy. In a retrospective cohort study, 332 patients with prostate cancer ISUP group of 2 or lower were included in confirmatory biopsy protocol using MRI FUS-TB. The protocol established performing MRI FUS-TB at 12–24 months. 11.7% of patients were upgraded to ISUP 3 during follow up and those with a PSA density ≥ 0.15 ng/mL were at higher risk for upgrade on confirmatory biopsy. Therefore, the combination of confirmatory biopsy with MRI and PSA density could be an interesting combination tool to explore in prospective trials [34]. Similarly, Lai et al. reported the results of a nomogram model in 76 patients that included clinical and imaging factors such as total lesion density, volume, and number of lesions, reporting an AUC of 0.84 to predict upgrading on confirmatory MRI FUS-TB on patients considered for active surveillance. Their results showed that 26.32% of patients were upgraded and the median specific antigen level was 5.1 ng/mL [35].

## 5. MRI and Artificial Intelligence

MRI in PCa is an evolving tool which has contributed to the field of diagnosis, staging, and determination of treatment options for this disease. However, a concerning limitation for wide implementation of this technique has been inter-reader variability and experience which is known to affect its performance [36,37,38,39]. AI based on the development and training of algorithms is being studied as a tool to overcome this limitation. Machine learning, a type of AI, is a technology characterized by learning with specific algorithms that can generate predictive models derived from data from different input sources. The input sources can be structured like data from clinical trials or unstructured data, and the learning abilities of machine learning can be divided into supervised or unsupervised categories. Supervised based algorithms models are preferably used when the training data that is generated as an input is associated with an objective and known outcome [40,41,42]. On the other hand, artificial neural networks are a subset of machine learning (ML) replicating the function of the inter-neuronal network of the human brain and are one of the most used models of machine learning in oncology [43,44,45].

AI in PCa has been applied in different settings such as assessment of benign/malignant lesions, to distinguish significant versus non-significant cancer, defining active surveillance candidates, staging, prediction of response to treatment, and recurrence among others [46,47,48,49,50]. In terms of diagnosis, many studies have reported the advantages of different AI models to provide accurate diagnosis of PCa [51,52,53,54,55,56].

Kwak et al. investigated the association between mpMRI and digital histopathologic analysis (DHA). They used machine learning models in 40 patients to compare the mpMRI and DHA. In their study, DHA correlated positively both with specific regions containing benign versus malignant tissues (*p* < 0.1), and with Gleason score in the transition zone [57]. Also, Khosravi et al. developed an AI based model for early diagnosis of PCa based on a retrospective study of 400 patients with mpMRI data sets and histological data. The authors reported an AUC of 0.89 (95% confidence interval 0.86–0.92) to distinguish between malignant versus benign lesions with an AUC of 0.78 for discriminating between high-risk versus low-risk PCa [58].

Similarly, a prospective study from Sweden included 6953 slides from men diagnosed with PCa and assessed the capacity of AI in predicting the presence, extension, and Gleason grade in the tissue samples. AI achieved an AUC of 0.997 to distinguish between benign and malignant lesions and a mean pairwise kappa of 0.62 for predicting Gleason grade. These data suggest that AI based on training algorithm can detect and grade cancer comparable to prostate cancer specialists [59]. 

Likewise, a single institution study of 316 men compared radiomic machine learning; mean apparent diffusion coefficient (ADC), and radiologic assessment for characterizing benign versus malignant lesions. Quantitative measurement of the ADC significantly had a 62% specificity and a 90% sensitivity which had similar performance with radiomic machine learning and outperformed clinical assessment [60].

Interestingly, Bulten et al. developed a deep-learning system to be able to reduce the inter-observer variability for determining Gleason grade. A total of 1243 patients were included in this retrospective study performed in the Netherlands in which 5759 biopsy specimens were collected. The deep learning automated system demonstrated an AUC of 0.990 for differentiating between benign and malignant lesions and outperformed 10 of the 15 pathologist in determining Gleason grade, with high agreement with the reference standard (quadratic Cohen’s kappa 0.918 95% CI 0.891–0.941) [61]. Furthermore, Pantanowitz et al. reported clinical validation of an algorithm for distinguishing between low-grade (Gleason score ≤ 6) and high-grade cancer (Gleason 7–10) with an AUC of 0.941 and described the first case of missed cancer detected by the model implemented [62]. Also, another study assessed the frequency of exact agreement of a deep learning system versus general pathologist, using the subspecialists (uropathologists) as a reference, when stratifying Gleason score in 752 biopsies. In this study the deep learning system showed a higher agreement rate of 71% vs. 58% for grading tumor biopsy. In terms of differentiating malignant from benign lesions, the agreement rate with subspecialists was not statistical different to that made by general pathologist [63]. Another study compared deep convolutional neural network (DCNN) to scale-invariant feature transform (non deep learning) in 172 patients. Deep learning had a statistically higher AUC than non-deep learning for detection of PCa, 0.84 and 0.70, respectively (*p* = 0.0007 < 0.001) [64].

PCa is an heterogenous disease in terms of aggressiveness and currently there are no effective biomarkers available to predict the nature and progression of the disease. A retrospective study of 64 patients with PCa found 14 radiomics features that correlated with the Gleason score and 31 histogram and texture characteristics that correlated with different gene signatures. Although prospective trials are required, radiomics features with machine learning prediction models are promising markers for cancer aggressiveness [65].

Another possible key application for AI models is to assist in reducing unnecessary biopsies in certain clinical scenarios to decrease the overload of healthcare systems, especially where specialists are not easily available [66,67]. A retrospective study of 346 men with PIRADS 3 lesions underwent mpMRI and transperineal MRI-US-targeted biopsy. PCa was detected in 20.6% of patients and 79% of men had benign lesions. Four different machine learning systems based on clinical factors such as age, prostate-specific antigen levels, imagenologic characteristics, and histopathology results were utilized and studied to be able to distinguish benign versus malignant lesions. The “Forrest Classifier”, which is a machine-learning model-based classifier, obtained the best performance with overall accuracy of 0.860 proving to be a useful tool in more efficient diagnostic decision making [68].

AI has also been studied in terms of response to treatment in patients with metastatic castration resistant prostate cancer. Deng et al. implemented a model based on 78 different features that included laboratory abnormalities, patient characteristics, and clinical and oncological history. Prediction performance was evaluated by area under the curve models in 1600 patients. Determination of 10 important features in an implemented model demonstrated that early discontinuation of treatment can be predicted from clinical information of the patients [69].

## 6. Conclusions

mpMRI technique has evolved in the past years from T1-weighted and T2-weighted sequences to modern sequences such as DWI, DCE, and magnetic resonance spectroscopy imaging which have improved the diagnosis of prostate cancer in the general population in multiple clinical scenarios [70,71,72,73,74]. As of today, mpMRI is suggested as a diagnostic and staging tool in most clinical guidelines as an aid in diminishing diagnosis of clinically insignificant tumors that could avoid aggressive unnecessary treatments, reducing psychological distress (anxiety, fear, and depression) as well as detection of the truly significant tumors whose prompt treatment will have an impact on mortality [66,67,75,76,77]. Although mp-MRI has some limitations, such as costs and variability due to inter-reader experience, promising technologies like artificial intelligence, as well as broader use worldwide, are attempting to contribute to overcome these limitations.

## Figures and Tables

**Table 1:** Comparison of prostate biopsy techniques.

	In Bore	Cognitive Biopsy	MRI–TRUS Fusion Biopsy
Cost	Higher	Lower	Higher
Duration < 30 min	No	Yes	No
Requires Software Platform	Yes	No	Yes
Operator Expertise	Yes	Yes	No
Target Small Lesions	Yes	No	Yes

**Table 2:** Prostate cancer detection rates.

	PROMIS^9^	PRECISION^10^
Number of Patients	740	500
Detection of PCa MRI Targeted Biopsy Group	40%	38%
Detection of PCa in Standard Biopsy Group	26%	26%
Sensitivity	93%	NR
Negative Predictive Value	89%	NR
Detection Rate of CS PCa	PIRADS 3: 21%	PIRADS 3: 29%
	PIRADS 4: 58%	PIRADS 4: 40%
	PIRADS 5: 81%	PIRADS 5: 31%

NR: not reported.

**Table 3:** Role of MRI in population of patients with a rising PSA and prior negative systematic TRUS guided prostate biopsy.

	PCa Detection Rates	High Grade PCa	Sensitivity	Negative Predictive Value
Abd-Alazzez et al.	59%	93%	76%	79%
Vourganti et al.	37%	11%	76%	NR
Wegelin et al.	35%	34.2%	NR	NR

NR: not reported.

**Table 4:** MRI for determining extracapsular extension and seminal vesical invasion.

	ECE	SVI
Sensitivity	54–87%	19–85%
Specificity	90–92%	90–100%
Positive predictive value	81–84%	80–100%
Negative predictive value	74–94%	76–96%
Dominguez et al. and Abdulin et al.		

## Data Availability

Not applicable.

## References

[1] Bray F., Me J.F., Soerjomataram I., Siegel R.L., Torre L.A., Jemal A. (2018). Global cancer statistics 2018: GLOBOCAN estimates of incidence and mortality worldwide for 36 cancers in 185 countries. CA Cancer J. Clin..

[2] Damadian R. (1971). Tumor Detection by Nuclear Magnetic Resonance. Science.

[3] Steyn J.H., Smith F.W. (1982). Nuclear Magnetic Resonance Imaging of the Prostate. BJU Int..

[4] Narayan P., Jajodia P., Kurhanewicz J., Thomas A., MacDonald J., Hubesch B., Hedgcock M., Anderson C.M., James T.L., Tanagho E.A. (1991). Characterization of prostate cancer, benign prostatic hyperplasia and normal prostates using transrectal 31phosphorus magnetic resonance spectroscopy: A preliminary report. J. Urol..

[5] Tamada T., Kido A., Takeuchi M., Yamamoto A., Miyaji Y., Kanomata N., Sone T. (2019). Comparison of PI-RADS version 2 and PI-RADS version 2.1 for the detection of transition zone prostate cancer. Eur. J. Radiol..

[6] Wegelin O., van Melick H.H., Hooft L., Bosch J.R., Reitsma H.B., Barentsz J.O., Somford D.M. (2017). Comparing Three Different Techniques for Magnetic Resonance Imag-ing-targeted Prostate Biopsies: A Systematic Review of In-bore versus Magnetic Resonance Imaging-transrectal Ultrasound fusion versus Cognitive Registration. Is There a Preferred Technique?. Eur Urol..

[7] Lowrance W.T., Breau R.H., Chou R., Chapin B.F., Crispino T., Dreicer R., Cookson M.S. (2021). Advanced Prostate Cancer: AUA/ASTRO/SUO Guideline PART I. J. Urol..

[8] Mohler J.L., Antonarakis E.S., Armstrong A.J., D’Amico A.V., Davis B.J., Dorff T., Eastham J.A., Enke C.A., Farrington T.A., Higano C.S. (2019). Prostate Cancer, Version 2.2019, NCCN Clinical Practice Guidelines in Oncology. J. Natl. Compr. Cancer Netw..

[9] Ahmed H.U., Bosaily A.E.-S., Brown L.C., Gabe R., Kaplan R.S., Parmar M.K., Collaco-Moraes Y., Ward K., Hindley R.G., Freeman A. (2017). Diagnostic accuracy of multi-parametric MRI and TRUS biopsy in prostate cancer (PROMIS): A paired validating confirmatory study. Lancet.

[10] Kasivisvanathan V., Rannikko A., Borghi M., Panebianco V., Mynderse L.A., Vaarala M., Briganti A., Budäus L., Hellawell G., Hindley R.G. (2018). MRI-Targeted or Standard Biopsy for Prostate-Cancer Diagnosis. N. Engl. J. Med..

[11] Ahdoot M., Wilbur A.R., Reese S.E., Lebastchi A.H., Mehralivand S., Gomella P., Bloom J., Gurram S., Siddiqui M., Pinsky P. (2020). MRI-Targeted, Systematic, and Combined Biopsy for Prostate Cancer Diagnosis. N. Engl. J. Med..

[12] Abd-Alazeez M., Ahmed H.U., Arya M., Charman S.C., Anastasiadis E., Freeman A., Emberton M., Kirkham A. (2013). The accuracy of multiparametric MRI in men with negative biopsy and elevated PSA level—Can it rule out clinically significant prostate cancer?. Urol. Oncol. Semin. Orig. Investig..

[13] Hambrock T., Somford D.M., Hoeks C., Bouwense S.A., Huisman H., Yakar D., Van Oort I.M., Witjes J.A., Fütterer J.J., Barentsz J.O. (2010). Magnetic Resonance Imaging Guided Prostate Biopsy in Men with Repeat Negative Biopsies and Increased Prostate Specific Antigen. J. Urol..

[14] Vourganti S., Rastinehad A., Yerram N.K., Nix J., Volkin D., Hoang A., Turkbey B., Gupta G.N., Kruecker J., Linehan W.M. (2012). Multiparametric Magnetic Resonance Imaging and Ultrasound Fusion Biopsy Detect Prostate Cancer in Patients with Prior Negative Transrectal Ultrasound Biopsies. J. Urol..

[15] Wegelin O., Exterkate L., van der Leest M., Kummer J.A., Vreuls W., de Bruin P.C., van Melick H.H. (2019). The FUTURE Trial: A Multicenter Randomised Controlled Trial on Target Biopsy Techniques Based on Magnetic Resonance Imaging in the Diagnosis of Prostate Cancer in Patients with Prior Neg-ative Biopsies. Eur. Urol..

[16] Goldberg H., Ahmad A.E., Chandrasekar T., Klotz L., Emberton M., Haider M.A., Taneja S., Arora K., Fleshner N., Finelli A. (2020). Comparison of Magnetic Resonance Imaging and Transrectal Ultrasound Informed Prostate Biopsy for Prostate Cancer Diagnosis in Biopsy Naïve Men: A Systematic Review and Meta-Analysis. J. Urol..

[17] Truong M., Wang B., Gordetsky J.B., Nix J.W., Frye T.P., Messing E.M., Thomas J.V., Feng C., Rais-Bahrami S. (2017). Multi-institutional nomogram predicting benign prostate pathology on magnetic resonance/ultrasound fusion biopsy in men with a prior negative 12-core systematic biopsy. Cancer.

[18] Volkin D., Turkbey B., Hoang A.N., Rais-Bahrami S., Yerram N., Walton-Diaz A., Nix J.W., Wood B., Choyke P.L., Pinto P.A. (2014). Multiparametric magnetic resonance imaging (MRI) and subsequent MRI/ultrasonography fusion-guided biopsy increase the detection of anteriorly located prostate cancers. BJU Int..

[19] Murphy I.G., Nimhurchu E., Gibney R.G., McMahon C.J. (2017). MRI-directed cognitive fusion-guided biopsy of the anterior prostate tumors. Diagn. Interv. Radiol..

[20] Diaz A.W., Hoang A.N., Turkbey B., Hong C.W., Truong H., Sterling T., Rais-Bahrami S., Siddiqui M., Stamatakis L., Vourganti S. (2013). Can Magnetic Resonance-Ultrasound Fusion Biopsy Improve Cancer Detection in Enlarged Prostates?. J. Urol..

[21] de Gorski A., Rouprêt M., Peyronnet B., Le Cossec C., Granger B., Comperat E., Mozer P. (2015). Accuracy of Magnetic Resonance Imaging/Ultrasound Fusion Targeted Biopsies to Diagnose Clinically Significant Prostate Cancer in Enlarged Compared to Smaller Prostates. J. Urol..

[22] Dominguez C., Plata M., Cataño J.G., Palau M., Aguirre D., Narvaez J., Trujillo S., Gómez F., Trujillo C.G., Caicedo J.I. (2018). Diagnostic accuracy of multiparametric magnetic resonance imaging in detecting extracapsular extension in intermediate and high-risk prostate cancer. Int. Braz. J. Urol..

[23] Abdullin I.I., Kossov F.A., Kamolov B.S., Kapustin V.V., Grigorev N.A., Gubskii I.L., Matveev V.B. (2018). Use of multiparametric magnetic resonance imaging in t-staging of prostate cancer: A multicenter study. Urologiia.

[24] Diamand R., Ploussard G., Roumiguié M., Oderda M., Benamran D., Fiard G., Quackels T., Assenmacher G., Simone G., Van Damme J. (2020). External Validation of a Multiparametric Magnetic Resonance Imaging–based Nomogram for the Prediction of Extracapsular Extension and Seminal Vesicle Invasion in Prostate Cancer Patients Undergoing Radical Prostatectomy. Eur. Urol..

[25] Diaz A.W., Shakir N.A., George A.K., Rais-Bahrami S., Turkbey B., Rothwax J.T., Stamatakis L., Hong C.W., Siddiqui M.M., Okoro C. (2015). Use of serial multiparametric magnetic resonance imaging in the management of patients with prostate cancer on active surveillance. Urol. Oncol. Semin. Orig. Investig..

[26] Komisarenko M., Martin L.J., Finelli A. (2018). Active surveillance review: Contemporary selection criteria, follow-up, compliance and outcomes. Transl. Androl. Urol..

[27] Walker C.H., Marchetti K.A., Singhal U., Morgan T.M. (2021). Active surveillance for prostate cancer: Selection criteria, guidelines, and outcomes. World J. Urol..

[28] Dall’Era M.A., Albertsen P.C., Bangma C., Carroll P.R., Carter H.B., Cooperberg M., Freedland S.J., Klotz L.H., Parker C., Soloway M.S. (2012). Active Surveillance for Prostate Cancer: A Systematic Review of the Literature. Eur. Urol..

[29] Sathianathen N.J., Konety B.R., Alarid-Escudero F., Lawrentschuk N., Bolton D.M., Kuntz K.M. (2019). Cost-effectiveness Analysis of Active Surveillance Strategies for Men with Low-risk Prostate Cancer. Eur. Urol..

[30] Klotz L., Zhang L., Lam A., Nam R., Mamedov A., Loblaw A. (2010). Clinical results of long-term follow-up of a large, active sur-veillance cohort with localized prostate cancer. J. Clin. Oncol..

[31] Turkbey B., Mani H., Aras O., Ho J., Hoang A., Rastinehad A.R., Agarwal H., Shah V., Bernardo M., Pang Y. (2013). Prostate cancer: Can multiparametric MR imaging help identify patients who are candidates for active surveillance?. Radiology.

[32] Muehlematter U.J., Burger I.A., Becker A.S., Schawkat K., Hötker A.M., Reiner C.S., Müller J., Rupp N.J., Rüschoff J.H., Eberli D. (2019). Diagnostic Accuracy of Multiparametric MRI versus 68Ga-PSMA-11 PET/MRI for Extracapsular Extension and Seminal Vesicle Invasion in Patients with Prostate Cancer. Radiology.

[33] Klotz L., Pond G., Loblaw A., Sugar L., Moussa M., Berman D., Haider M. (2020). Randomized Study of Systematic Biopsy Versus Magnetic Resonance Imaging and Tar-geted and Systematic Biopsy in Men on Active Surveillance (ASIST): 2-year Postbiopsy Follow-up. Eur. Urol..

[34] Jayadevan R., Felker E.R., Kwan L., Barsa D.E., Zhang H., Sisk A.E., Delfin M., Marks L.S. (2019). Magnetic Resonance Imaging–Guided Confirmatory Biopsy for Initiating Active Surveillance of Prostate Cancer. JAMA Netw. Open.

[35] Lai W.S., Gordetsky J.B., Thomas J.V., Nix J.W., Rais-Bahrami S. (2017). Factors predicting prostate cancer upgrading on magnetic resonance imaging-targeted biopsy in an active surveillance population. Cancer.

[36] Brembilla G., Dell’Oglio P., Stabile A., Damascelli A., Brunetti L., Ravelli S., Cristel G., Schiani E., Venturini E., Grippaldi D. (2020). Interreader variability in prostate MRI reporting using Prostate Imaging Reporting and Data System version 2.1. Eur. Radiol..

[37] Girometti R., Giannarini G., Greco F., Isola M., Cereser L., Como G., Sioletic S., Pizzolitto S., Crestani A., Ficarra V. (2018). Interreader agreement of PI-RADS v. 2 in assessing prostate cancer with multiparametric MRI: A study using whole-mount histology as the standard of reference. J. Magn. Reson. Imaging.

[38] Ba C.P.S., Harmon S.A., Barrett T., Bittencourt L.K., Law Y.M., Shebel H., An J.Y., Czarniecki M., Mehralivand S., Coskun M. (2018). Intra- and interreader reproducibility of PI-RADSv2: A multireader study. J. Magn. Reson. Imaging.

[39] Rosenkrantz A., Lim R.P., Haghighi M., Somberg M., Babb J.S., Taneja S.S. (2013). Comparison of Interreader Reproducibility of the Prostate Imaging Reporting and Data System and Likert Scales for Evaluation of Multiparametric Prostate MRI. Am. J. Roentgenol..

[40] Buch V.H., Ahmed I., Maruthappu M. (2018). Artificial intelligence in medicine: Current trends and future possibilities. Br. J. Gen. Pr..

[41] Letzen B., Wang C.J., Chapiro J. (2018). The Role of Artificial Intelligence in Interventional Oncology: A Primer. J. Vasc. Interv. Radiol..

[42] Harmon S.A., Tuncer S., Sanford T., Choyke P.L., Turkbey B. (2019). Artificial intelligence at the intersection of pathology and radiology in prostate cancer. Diagn. Interv. Radiol..

[43] Egevad L., Ström P., Kartasalo K., Olsson H., Samaratunga H., Delahunt B., Eklund M. (2020). The utility of artificial intelligence in the assessment of prostate pathology. Histopathology.

[44] Nagy M., Radakovich N., Nazha A. (2020). Machine learning in oncology: What should clinicians know?. JCO Clin. Cancer Inform..

[45] Hu X., Cammann H., Meyer H.-A., Miller K., Jung K., Stephan C. (2013). Artificial neural networks and prostate cancer—Tools for diagnosis and management. Nat. Rev. Urol..

[46] Checcucci E., Autorino R., Cacciamani G.E., Amparore D., De Cillis S., Piana A., Piazzolla P., Vezzetti E., Fiori C., Veneziano D. (2020). Artificial intelligence and neural networks in urology: Current clinical applications. Minerva Urol Nefrol..

[47] Anagnostou T., Remzi M., Lykourinas M., Djavan B. (2003). Artificial Neural Networks for Decision-Making in Urologic Oncology. Eur. Urol..

[48] Shah M., Naik N., Somani B.K., Hameed B.M.Z. (2020). Artificial intelligence (AI) in urology-Current use and future directions: An iTRUE study. Turk. J. Urol..

[49] Hameed B., Dhavileswarapu A.S., Raza S., Karimi H., Khanuja H., Shetty D., Ibrahim S., Shah M., Naik N., Paul R. (2021). Artificial Intelligence and Its Impact on Urological Diseases and Management: A Comprehensive Review of the Literature. J. Clin. Med..

[50] Suarez-Ibarrola R., Sigle A., Eklund M., Eberli D., Miernik A., Benndorf M., Bamberg F., Gratzke C. (2021). Artificial Intelligence in Magnetic Resonance Imaging–based Prostate Cancer Diagnosis: Where Do We Stand in 2021?. Eur. Urol. Focus.

[51] Tătaru O., Vartolomei M., Rassweiler J., Virgil O., Lucarelli G., Porpiglia F., Amparore D., Manfredi M., Carrieri G., Falagario U. (2021). Artificial Intelligence and Machine Learning in Prostate Cancer Patient Management—Current Trends and Future Perspectives. Diagnostics.

[52] Cimadamore A., Cheng L., Scarpelli M., Lopez-Beltran A., Montironi R. (2021). Digital diagnostics and artificial intelligence in prostate cancer treatment in 5 years from now. Transl. Androl. Urol..

[53] Schreiber A., Hahn H., Wenzel M., Loch T. (2020). Künstliche Intelligenz. Der. Urol..

[54] Shimizu H., Nakayama K.I. (2020). Artificial intelligence in oncology. Cancer Sci..

[55] Bera K., Schalper K.A., Rimm D.L., Velcheti V., Madabhushi A. (2019). Artificial intelligence in digital pathology—New tools for diagnosis and precision oncology. Nat. Rev. Clin. Oncol..

[56] Cuocolo R., Caruso M., Perillo T., Ugga L., Petretta M. (2020). Machine Learning in oncology: A clinical appraisal. Cancer Lett..

[57] Suarez-Ibarrola R., Hein S., Reis G., Gratzke C., Miernik A. (2019). Current and future applications of machine and deep learning in urology: A review of the literature on urolithiasis, renal cell carcinoma, and bladder and prostate cancer. World J. Urol..

[58] Wang X., Yang W., Weinreb J., Han J., Liang W., Kong X., Yan Y., Ke Z., Luo B., Liu T. (2017). Searching for prostate cancer by fully automated magnetic resonance imaging classification: Deep learning versus non-deep learning. Sci. Rep..

[59] Kwak J.T., Sankineni S., Xu S., Turkbey B., Choyke P.L., Pinto P.A., Moreno V., Merino M., Wood B.J. (2017). Prostate Cancer: A Correlative Study of Multiparametric MR Imaging and Digital Histopathology. Radiology.

[60] Ström P., Kartasalo K., Olsson H., Solorzano L., Delahunt B., Berney D.M., Bostwick D.G., Evans A.J., Grignon D.J., Humphrey P.A. (2020). Artificial intelligence for diagnosis and grading of prostate cancer in biopsies: A population-based, diagnostic study. Lancet Oncol..

[61] Bonekamp D., Kohl S., Wiesenfarth M., Schelb P., Radtke J.P., Goetz M., Kickingereder P.V., Yaqubi K., Hitthaler B., Gählert N. (2018). Radiomic Machine Learning for Characterization of Prostate Lesions with MRI: Comparison to ADC Values. Radiology.

[62] Bulten W., Pinckaers H., van Boven H., Vink R., de Bel T., van Ginneken B., van der Laak J., de Kaa C.H.-V., Litjens G. (2020). Automated deep-learning system for Gleason grading of prostate cancer using biopsies: A diagnostic study. Lancet Oncol..

[63] Pantanowitz L., Quiroga-Garza G.M., Bien L., Heled R., Laifenfeld D., Linhart C., Sandbank J., Shach A.A., Shalev V., Vecsler M. (2020). An artificial intelligence algorithm for prostate cancer diagnosis in whole slide images of core needle biopsies: A blinded clinical validation and deployment study. Lancet Digit. Health.

[64] Nagpal K., Foote D., Tan F., Liu Y., Chen P.-H.C., Steiner D.F., Manoj N., Olson N., Smith J.L., Mohtashamian A. (2020). Development and Validation of a Deep Learning Algorithm for Gleason Grading of Prostate Cancer from Biopsy Specimens. JAMA Oncol..

[65] Khosravi P., Bs M.L., Eljalby M., Li Q., Kazemi E., Ms P.Z., Ms A.S., Brendel M., Barnes J., Ricketts C. (2021). A Deep Learning Approach to Diagnostic Classification of Prostate Cancer Using Pathology–Radiology Fusion. J. Magn. Reson. Imaging.

[66] Hectors S., Cherny M., Yadav K.K., Beksaç A.T., Thulasidass H., Lewis S., Davicioni E., Wang P., Tewari A.K., Taouli B. (2019). Radiomics Features Measured with Multiparametric Magnetic Resonance Imaging Predict Prostate Cancer Aggressiveness. J. Urol..

[67] Turkbey B., Brown A.M., Sankineni S., Wood B., Pinto P.A., Choyke P.L. (2015). Multiparametric prostate magnetic resonance imaging in the evaluation of prostate cancer. CA A Cancer J. Clin..

[68] Turkbey B., Choyke P.L. (2014). A decade in image-guided prostate biopsy. Nat. Rev. Urol..

[69] Kan Y., Zhang Q., Hao J., Wang W., Zhuang J., Gao J., Huang H., Liang J., Marra G., Calleris G. (2020). Clinico-radiological characteristic-based machine learning in reducing unnecessary prostate biopsies of PI-RADS 3 lesions with dual validation. Eur. Radiol..

[70] Deng K., Li H., Guan Y. (2019). Treatment Stratification of Patients with Metastatic Castration-Resistant Prostate Cancer by Machine Learning. iScience.

[71] Stabile A., Giganti F., Emberton M., Moore C.M. (2018). MRI in prostate cancer diagnosis: Do we need to add standard sampling? A review of the last 5 years. Prostate Cancer Prostatic Dis..

[72] Monni F., Fontanella P., Grasso A., Wiklund P., Ou Y.-C., Randazzo M., Rocco B., Montanari E., Bianchi G. (2017). Magnetic resonance imaging in prostate cancer detection and management: A systematic review. Minerva Urol. Nefrol. Ital. J. Urol. Nephrol..

[73] Bittencourt L.K., De Hollanda E.S., De Oliveira R.V. (2016). Multiparametric MR Imaging for Detection and Locoregional Staging of Prostate Cancer. Top. Magn. Reson. Imaging.

[74] Rais-Bahrami S., Siddiqui M., Turkbey B., Stamatakis L., Logan J., Hoang A.N., Walton-Diaz A., Vourganti S., Truong H., Kruecker J. (2013). Utility of Multiparametric Magnetic Resonance Imaging Suspicion Levels for Detecting Prostate Cancer. J. Urol..

[75] Rosenkrantz A.B., Taneja S.S. (2015). Prostate MRI Can Reduce Overdiagnosis and Overtreatment of Prostate Cancer. Acad. Radiol..

[76] Thompson J., Lawrentschuk N., Frydenberg M., Thompson L., Stricker P. (2013). Usanz The role of magnetic resonance imaging in the diagnosis and management of prostate cancer. BJU Int..

[77] Holtz J.N., Tay K.J., Polascik T.J., Gupta R.T. (2016). Integration of multiparametric MRI into active surveillance of prostate cancer. Futur. Oncol..

